# Dandelion‐Inspired, Wind‐Dispersed Polymer‐Assembly Controlled by Light

**DOI:** 10.1002/advs.202206752

**Published:** 2022-12-27

**Authors:** Jianfeng Yang, Hang Zhang, Alex Berdin, Wenqi Hu, Hao Zeng

**Affiliations:** ^1^ Faculty of Engineering and Natural Sciences Tampere University P.O. Box 541 Tampere FI‐33101 Finland; ^2^ Department of Applied Physics Aalto University P.O. Box 15100 Espoo FI‐02150 Finland; ^3^ Max Planck Institute for Intelligent Systems, Stuttgart 70569 Stuttgart Germany

**Keywords:** dispersal, light‐driven, liquid crystal elastomer, passive flier, separated vortex ring, soft actuator

## Abstract

The rise of stimuli‐responsive polymers has brought about a wealth of materials for small‐scale, wirelessly controlled soft‐bodied robots. Thinking beyond conventional robotic mobilities already demonstrated in synthetic systems, such as walking, swimming and jumping, flying in air by dispersal, gliding, or even hovering is a frontier yet to be explored by responsive materials. The demanding requirements for actuator's performance, lightweight, and effective aerodynamic design underlie the grand challenges. Here, a soft matter‐based porous structure capable of wind‐assisted dispersal and lift‐off/landing action under the control of a light beam is reported. The design is inspired by the seed of dandelion, resembling several biomimetic features, i.e., high porosity, lightweight, and separated vortex ring generation under a steady wind flow. Superior to its natural counterparts, this artificial seed is equipped with a soft actuator made of light‐responsive liquid crystalline elastomer, which induces reversible opening/closing actions of the bristles upon visible light excitation. This shape‐morphing enables manual tuning of terminal velocity, drag coefficient, and wind threshold for dispersal. Optically controlled wind‐assisted lift‐off and landing actions, and a light‐induced local accumulation in descending structures are demonstrated. The results offer novel approaches for wirelessly controlled, miniatured devices that can passively navigate over a large aerial space.

## Graphical abstract

Wind‐assisted dispersal is widely used by natural plants to spread their seeds for sowing. Here, a passive disperser inspired by the bristly architecture of the seed of dandelion is reported. The disperser can perform light‐controlled shape‐morphing, separated vortex ring generation, lift‐off and landing actions, self‐oscillation, and light‐induced spatial accumulation within the wind flow.

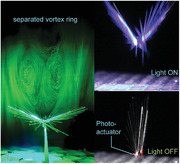

## 1 Introduction

Stimuli‐responsive shape‐morphing polymers are synthetic materials capable of programmable deformation upon external stimuli.^[^ ^1^, ^2^ ^]^ Various types of stimuli have been successfully implemented, such as heat, humidity, chemical reaction, magnetic, electric, and light fields,^[^ ^3^, ^4^, ^5^, ^6^, ^7^, ^8^ ^]^ to produce versatile shape‐morphing capabilities (bending, twisting, contracting‐expanding, buckling, etc.) in soft systems.^[^ ^9^, ^10^ ^]^ These intriguing shape‐changing abilities, often dictated by phase transition properties of soft matter,^[^ ^11^, ^12^ ^]^ offer a wealth of actuation opportunities for realizing miniature robots that are agile, externally controlled, untethered, and adaptive to the variation of the surrounding environment.^[^ ^13^ ^]^ Driven by the long‐term vision to revolutionize medical treatments and carry out robotic execution within constrained or hazard environments, responsive materials have been developed into swimmers to translocate through bio‐fluidics‐like channels,^[^ ^14^ ^]^ walkers for cargo transportation and assembly,^[^ ^15^ ^]^ rollers and jumpers for instant movements.^[^ ^16^ ^]^ However, flying – locomotion through a spatial volume, concerning actions such as wind‐assisted dispersal and gliding, take‐off and hovering through active mechanical movements – is one of the most challenging frontiers yet to be explored in responsive material research.

The realization of a flying object at a small scale (i.e., less than ten centimeters) requires the ultra‐high performance of actuator materials. To date, piezoelectric,^[^ ^17^ ^]^ electromagnetic actuators,^[^ ^18^ ^]^ and dielectric elastomers^[^ ^19^, ^20^ ^]^ have been utilized for such purposes. These materials possess high power density (> 200 W kg^−1^), high bandwidth (> 100 Hz) and good compatibility with control circuits and robotic skeletons. However, they all rely on an electric cable connection for powering, either in an on‐board or off‐board manner. Currently, it is still impossible to use responsive materials to achieve wirelessly controlled, actively flying objects, due to the limitation of these materials’ properties. For instance, responsive materials with best‐performing high active stress (> 10 MPa) and energy density (> 500 KJ m^−3^) are slow to respond.^[^ ^21^, ^22^, ^23^ ^]^ By scaling down the physical dimensions,^[^ ^24^ ^]^ some materials can achieve comparable power density as electrically driven ones, however, they fail in other aspects deemed crucial for an aerial robot, such as efficient integration with wings and hinges. Hence, realizing a micro‐scale active flier based on responsive materials is still a forbidden challenge for the current technique.

The wind‐dispersed seeds in nature have suggested another locomotion mode based on a passive flight mechanism.^[^ ^25^, ^26^ ^]^ Plants often adopt bristle bundle (pappus) or membrane wings to enhance the air drag on their seeds, prolonging the descent time that ensures a large dispersal distance assisted by the wind gust.^[^ ^27^ ^]^ These natural designs have inspired artificial constructs for the generation of vortex and the study of aerodynamic stability,^[^ ^28^ ^]^ as well as the fabrication of devices for passively distributing micro‐electronics.^[^ ^27^, ^29^ ^]^ Besides, there exist reports about gliding flight of a heat‐induced shape‐memory origami structure^[^ ^30^ ^]^ and tunability of gliding angle upon the photomechanical deformation of a polymer‐based wing.^[^ ^31^ ^]^ These seminal studies uncovered the possibility of effective interaction between airflow and an artificially engineered structure that can be controlled by using external stimuli. Herein, we hypothesize that the combination between miniature dispersal architecture and light‐induced deformation can yield a stimulus‐control performance of the passive flight (dispersal and gliding).

To verify the above hypothesis, we devised a soft structure composed of a bundle of bristles inspired by the seed of dandelion. A photomechanical actuator made of liquid crystalline elastomer (LCE) bending strip enables light‐controlled opening/closure of the pappus structure. We demonstrate optically triggered lift‐off and landing actions in individual structure under the assistance of wind flow, and light influence on the trajectory among multiple free‐descending objects.

## 2 Results

### 2.1 System Concept

**Figure 1:**
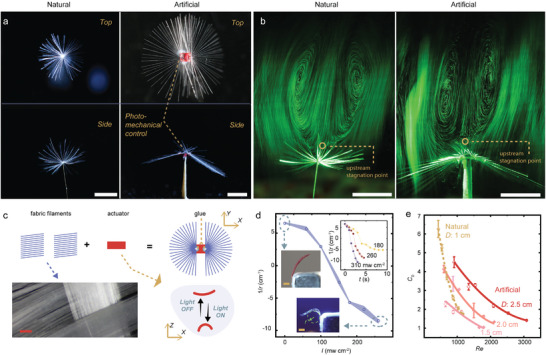
Dandelion‐inspired disperser. a) Top‐views and side‐view photos of natural dandelion and artificial disperser. b) Photographs of the separated vortex ring pattern created by natural dandelion seed and polymer assembly. All scale bars in (a,b) are 5 mm. c) Schematics of the fabrication process. Insets: photographic images of fabric filaments (left). Schematic drawing of light induced bending of the soft actuator (right). d) Change of strip curvature 1/*r* upon different light intensities. *r* is the radius of the bending arc. Inset: Photos of strip curvature change upon different light intensities (left). The kinetics of light induced deformation (right). Strip size for material characterization: 0.6 × 0.2 × 0.005 cm^3^. Experiments are performed without wind flow. Scale bar in (c,d) are 1 mm. e) Drag coefficient *C*_D_ of natural dandelion seed and artificial structures as a function of *Re* (Reynolds number). The error bars indicate standard deviation for *n* = 3 measurements.

Dandelions, the herbs commonly found on soil land all over the world, possess one of the most remarkable skills of dispersal among plant kingdoms. They are able to deliver their seeds by using updrafts and winds, across a range of over a hundred kilometers.^[^ ^28^ ^]^ **Figure** **1**a shows the architecture of a dandelion seed: it grows into a parachute‐like shape (pappus), containing a bundle of bristle filaments, each about 14 microns thick (Figure S1, Supporting Information). This specific bristly architecture brings about structural porosity (the ratio of the projected empty area to the pappus cross section, 0.92 in natural seed) ^[^ ^28^ ^]^ that results in an ultra‐low mass (≈0.73 mg of the entire seed, 0.15 mg of the pappus; *n* = 10 seeds) and air drag enhancement due to the wall effect^[^ ^32^ ^]^ around thick air boundaries between two filaments.

Inspired by the seed of the dandelion, we have fabricated a passive wind disperser by UV‐curing 54 pieces of bristly filament centrosymmetrically around a rectangular soft actuator film on a 2D plane (Figure 1c). The bristles are trimmed from a textile fabric (inset of Figure 1c), possessing a comparable diameter to the ones of the natural seed pappus. The comparison between the seed bristles and the fabric, see the meso‐to‐micro‐scale photographs in Figure S1 (Supporting Information). In our design, the filaments are organized into two groups, fixed on the opposite sides of the actuator strip, respectively (porosity is 0.95). To obtain mechanical actuation, we adopt chain‐extended LCE^[^ ^33^ ^]^ as the central active component for shape‐morphing. This LCE is synthetized by Aza‐Michael addition reaction,^[^ ^34^ ^]^ as often used in the state‐of‐the‐art LCE‐based robotics.^[^ ^35^, ^36^ ^]^ A splayed alignment and a light absorbing dye (Dispersed Red 1) are used to ensure a bending deformation^[^ ^37^ ^]^ upon illumination (schematic inset in Figure 1c). By changing the illuminating light intensity, different curvatures in LCE shape‐morphing are obtained (Figure 1d). The strip also shows an intensity dependent response in bending speed, as shown in the inset data of Figure 1d, the actuator has shortened the response from 7 to 2 s upon elevating the intensity from 180 to 310 mW cm^−2^. Figure S2 (Supporting Information) shows the chemical composition and the fabrication steps of the LCE. For details on material fabrication, and structural assembly, see in Experimental Section.

Separated vortex ring (SVR) patterns stabilized by the porosity gradient are observed downstream of a dandelion seed and the artificial assembly. The vortex ring exhibits a pair of air bubble patterns in the wake, detached from the seed body, as shown by the particle image velocimetry (PIV) images in Figure 1b, SVR stability in Figure S3 and Movie S1 (Supporting Information). A speed‐controlled wind tunnel was constructed, and a humidifier was used to seed the air in order to visualize the airflow through excitation of a laser sheet. Details of the wind tunnel construction and PIV measurement, see the Methods in Supporting Information. In our measurement, PIV data shows a reverse flow of about 10% of wind speed *U* occurs in both natural and artificial structures and the stagnation points of the upstream (z_su_, determined by the position of stream where a zero velocity is observed) always > 0 in both structures (Figure 1b). Such SVR formation is deemed important for passive dispersal mechanism^[^ ^28^ ^]^ in both natural structure and artificial assembly. Details of stagnation point positions and air flow velocity distribution downstream of the structures, see in the Supporting Information.

The terminal free‐falling velocity *U_t_* is measured to be 45.8, 48.7, 65.6 cm s^−1^ in artificial structures of 2.5, 2, and 1.5 cm size, compared to 47.0 cm s^−1^ in natural seeds (≈1 cm size diameter) under stationary air condition. The drag force^[^ ^38^ ^]^ can be determined by *F* = 0.5*C*_D_ *ρU* ^2^ *A*, where *C*_D_ is the drag coefficient, *ρ* the air density (1.29 g m^−3^), and *A* is the area of the structure. During the steady descent, *F* equals the gravitational force *G*, which is the product of mass (*m*) and acceleration constant (*g* = 9.8 m s^−2^). Trimming the seed segment (reduce of mass) and adding mass onto the structures result in different *U*_t_ (Figure S4, Supporting Information). Therefore, *C*_D_ can be calculated by measuring the terminal speed and the mass. To unveil the ratio of inertial to viscous forces inside the wind flow, Reynolds number^[^ ^39^ ^]^ *Re* (*UD/v*, where *D* is diameter, *v* is the kinematic viscosity of the air, 1.5 × 10^−5^ m^2^ s^−1^; *Re* ∝ *U*) is also calculated. *C*_D_ for natural seeds and artificial structures as a function of *Re* is plotted in Figure 1e. For *Re* < 700, natural pappus possess about double amount of drag force per unit area compared to the artificial structures, as a consequence of the naturally optimized three‐dimensional bristles that significantly decrease the air flow permeability. Conversely, for *Re* > 1000, the artificial structures deliver higher drag per unit area than the natural ones. We ascribe this to the unsteady vortex shedding at high *Re* that would reduce the *C*_D_ more significantly in natural pappus with a relatively smaller size.

### 2.2 Light Induced Shape‐Change in Polymer Assembly

**Figure 2:**
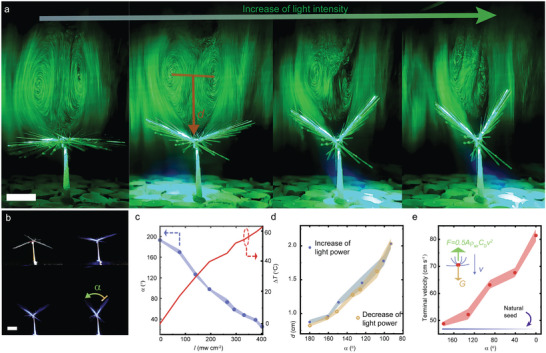
Photomechanical actuation in the artificial assembly. a) Photographs of the vortex ring pattern change of an artificial structure upon light illumination (left to right, 100, 150, 200, 300 mW cm^−2^). b) Side‐view photos of geometry changing upon light illumination. c) The change of open‐angle *α* and the corresponding temperature change Δ*T* as a function of light intensity *I*. d) The cyclic curve of the mean distance, *d*, from the vortex ring center to the structure as a function of *α*. e) The variation of terminal velocity of the structure with *α*. All structures are 2 cm in diameter. The error bars indicate the standard deviation for *n* = 3 measurements. LCE dimension: 4 mm × 2 mm × 0.005 mm. All scale bars are 0.5 cm.

**Figure 3:**
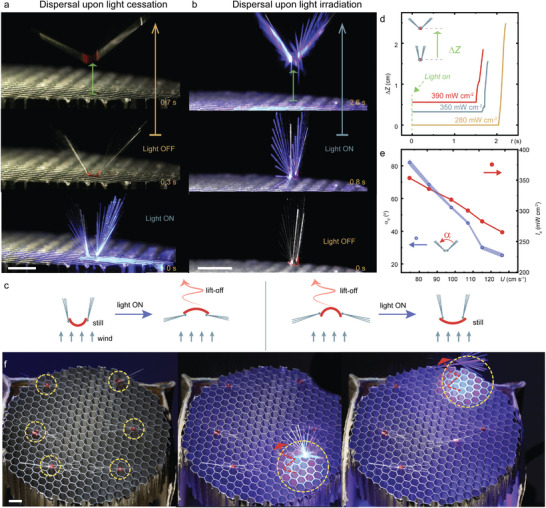
Light controlled lift‐off and landing. Series of photographs showing the passive dispersal of structures upon light cessation a) and light irradiation b). Wind flow: a) 68 cm s^−1^, b) 98 cm s^−1^. c) Schematic of geometries of the integral structure for light‐OFF dispersal and light‐ON dispersal. d) The change of vertical position ΔZ upon different light intensity illumination. e) The measured minimum open‐angle *α*_d_ for lift‐off action and the corresponding light intensity. f) The snapshot images of selected assemblies to lift‐off. Wind flow: 98 cm s^−1^. Light: 460 nm, 350 mW cm^−2^. LCE dimension: 4 mm × 2 mm × 0.005 mm. All scale bars are 0.5 cm.

We next implement the photomechanical actuation^[^ ^40^ ^]^ to fold/unfold the bristly structure, in order to manipulate the vortex formation downstream. Upon visible light excitation, the structure transforms from a nearly plane geometry into a V‐shape with an open‐angle *α* between two bristly wings (side‐view images in **Figure** **2**a,b, top‐view photos in Figure S5, Supporting Information). The folding of the structure is sensitive to impinged light intensity (*I*), as shown by the change of *α* as a function of *I* in Figure 2c (blue dots). Since the photothermal effect is responsible for the LCE bending, temperature change is also observed during the shape‐morphing (Figure 2c, red line). Details of photothermal kinetics against the air drag inside a wind tunnel with varying flow speeds is given in Figure S6 (Supporting Information). Upon shape‐morphing, the vortex ring pattern is distorted, as visualized in Figure 2a. This distortion (from flat to V shape) brings about three consequences. First, the structural asymmetry increases due to the fabrication/actuation imperfection and thus an enhance of vortex instability. Second, the mean distance between the vortex ring and the structure centers (*d*, as indicated in Figure 2a) increases. Third, the cross section of the structure decreases (Figure S5, Supporting Information). All these factors lead to a drag reduction upon folding of the structure. Hence, variation of the open‐angle effectively results in different *U_t_* (terminal velocities) for the descent. Figure 2e shows the *U_t_* measurement of several bristly constructs with different manually fixed angles under stationary air conditions, revealing that *U_t_* is increased by about a factor of 1.6 by folding the structure from 180° to 0°. Importantly, the shape‐change of the assembly is reversible and the distortion and re‐formation of the vortex ring can be reversibly controlled by photo‐induced folding and unfolding of the structure, as shown by the reversal of *d* during one actuation‐relaxation cycle (Figure 2d). This implies that the minimum magnitude of updraft wind for lift‐off action (the thresholding) can be fine‐tuned by light, and one can devise a light‐sensitive system to lift off or land under steady wind flow by using photomechanical control of the deformation (**Figure** **3**).

### 2.3 Light Controlled Dispersal

As discussed above, a folded structure requires a higher wind speed to initiate the lift‐off action than an unfolded one. Therefore, we place a light‐folded object inside a wind tunnel with flow velocity (68 cm s^−1^) just above the lift‐off wind threshold for an unfolded one (48 cm s^−1^), but below the threshold of the folded one (80 cm s^−1^). After ceasing the light, the structure quickly unfolds and takes off inside the wind flow (Figure 3a; Movie S2, Supporting Information). This passive dispersal upon light cessation (light‐OFF‐to‐disperse; light‐ON‐to‐land) is particularly intriguing for light induced landing with potential for optical collection over a large spatial volume, which will be elaborated later. On the other hand, we demonstrate the possibility of reversed dispersal control, where the light irradiation causes the lift‐off action (light‐ON‐to‐disperse and light‐OFF‐to‐land). In this case, an originally folded structure opens its bristly wings to lift off once a light beam is impinging onto the actuator (Figure 3b; Movie S2, Supporting Information). This is achieved by reverting the actuator film (bending direction) during fabrication. The structures of both control systems are schematically shown in Figure 3c, with more details in Figure S7 (Supporting Information). Figure 3d shows the tracking of vertical position change of the structure after switching on the light source, which exhibits a faster response with increasing intensity. Notice that, within a steady wind flow, there exists a minimum light induced open‐angle *α*_d_ to yield the detachment from the ground (lift‐off action), which is determined by the wind velocity, as depicted in Figure 3e.

The light‐ON‐to‐disperse strategy makes selective dispersal flight possible through localized illumination of individual seeds. Figure 3f and Movie S3 (Supporting Information) show a set of six structures placed on top of a wind tunnel (*U*: 98 cm s^−1^). All the structures are initially folded and thus remain still. A LED light beam is used to selectively activate the ones located at the right bottom corner and right upper corner, respectively. The selected structures have thus taken off and moved out from their initial position.

### 2.4 Light Influence on Flight Behavior over a Spatial Volume

**Figure 4:**
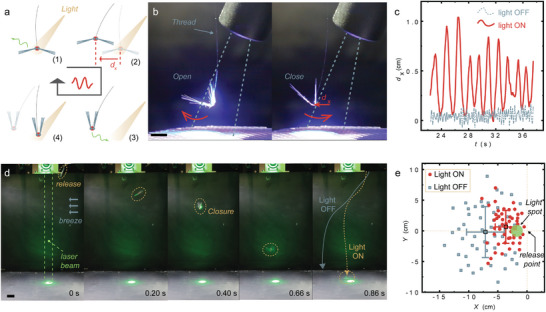
Light induced self‐oscillation and local accumulation of descending structures. a) Schematic of feedback design of a tethered self‐oscillating structure. b) Photos of oscillation around the light beam through opening and closure of the wings. Scale bar: 0.5 cm. Light: 460 nm, LED source, 210 W cm^−2^. LCE dimension: 4 mm × 2 mm × 0.005 mm. c) The data of periodic movement under light irradiation and the random fluctuation of the same structure without light irradiation, respectively. d) The descent trajectory of structures with laser ON and OFF. The vertical laser beam is set at 2 cm away from the releasing spot on the lateral plane (X–Y). Scale bar: 2 cm. Light spot: 532 nm, spot size: 2 cm, 1 W cm^−2^. e) The landing spot distribution on the X–Y plane when laser is ON (red dots) and OFF (blue squares).

The above studies demonstrated the light controlled lift‐off/landing action in a passive polymer assembly under the assistance of a steady wind flow. In the following, we offer more opportunities for manipulating the motion over spatial volume by using light‐ON‐to‐disperse strategy. **Figure** **4**a shows a self‐oscillating structure hanging by a thin thread (a single fabric filament) inside a wind tunnel with constant velocity, based on a feedback design. The feedback is established by using a constant illuminating light beam, around which an originally folded structure (light OFF state) unfolds the bristly wings when entering the light beam area (step 1, Figure 4a). The opening of wings increases the air drag exerted on the structure, which further drags the structure to swing out of the light area (step 2). LCE relaxes in the dark and recovers the structure to the folded geometry, which reduces air drag. This causes a backward swing into the light area as negative feedback to the motion (step 3). After returning to the illuminated area, the wings open again, and a new cycle starts (step 4 to 1). Figure 4b and Movie S4 (Supporting Information) show the oscillation around the light beam through opening and closure of the wings. Figure 4c plots the tracking data of lateral displacement (*d_x_*) of the geometrical center of the structure. By turning on the light source, the hanging structure exhibits a significantly enhanced magnitude of vibration and a clear periodicity (≈1.2 s) compared to the light‐OFF structure, of which movement is governed by the random fluctuations of the wind flow. The structure is able to perform oscillation as long as the material deformability remains functional (data for a longer time span, see Figure S8b, Supporting Information). In each oscillation cycle, there exists a positive correlation between the maximum open‐angle *α*_max_ (dictating the magnitude of the air drag) and the swing distance (oscillation amplitude) (Figure S8c, Supporting Information), indicating a photo‐deformation‐drag feedback mechanism. Such feedback mechanism offers an opportunity to dynamically localize a passive structure within the pre‐designed area in space through a light beam excitation and a tethered connection.

For passive flight over a large spatial area, it is interesting to explore the possibility of using light to control the gliding trajectory. This can open novel opportunities for manual collection of micro‐devices that have been dispersed in the open air. For the proof of principle, we adopt the light‐ON‐to‐land control strategy (as demonstrated in Figure 3a) and perform a free‐descent experiment of a structure multiple times. A structure is manually set at a position 30 cm above the ground and released for a descent under the vertical gravitational force, with the assistance of a slight horizontal wind flow (≈5 cm s^−1^). During the free‐drops without illumination, the structure shows a random distribution of trajectories (X–Z plane, Figure S9a, Supporting Information) and scattered landing points (X–Y plane, Figure 4e, blue squares). By applying a vertical light beam that illuminates a cylindrical volume (≈2 cm in diameter) in front of the releasing point, the structure will quickly fold the wings when passing through the optical beam volume (Figure 4d; Movie S5 in Supporting Information). This modifies the descending path of the structure and causes accumulation of landing spots close to the beam, as shown by a set of free‐drop experiment by releasing one identical structure 50 times from the same position (Figure 4e, red dots). Detailed statistical data about landing spot analysis, exemplified trajectories of free‐dropping structures with light ON and OFF conditions, see Figures S9 and S10 (Supporting Information).

## 3 Discussion

Control of the responsive material‐based disperser (or glider) over a large aerial space require long‐distance transmission of energy through the stimulus fields. Conventionally used stimulation, such as magnetic,^[^ ^7^ ^]^ electric fields,^[^ ^24^ ^]^ and chemical reaction,^[^ ^41^ ^]^ though succeeded in realizing versatile micro‐/nano‐ robots, are not accessible for a large spatial volume. Light stimuli (LED or laser sources), as demonstrated in this study, provide a feasible approach to introduce effective structural morphing for robotic motion while operating at long distances. The working distance of light‐controlled systems can be potentially extended to hundreds of meters or more by collimating a laser beam to be targeted on any objects. Thus, we believe the ultra‐long working distance together with separated control of multiple robots is the unique and superior capabilities of the light driven method compared to other stimulating sources. Future research may consider the improvement of photoactuation efficiency. With a reduced illumination intensity, i.e., < 100 mW cm^−2^ (one Sun),^[^ ^5^ ^]^ it is expected to yield other sophisticated functions, such as control of flight by the natural light, and adaptive dispersal real‐time influenced by sunlight shadow.

Another stimulus source deemed effective in material actuation and promising to access to large spaces is humidity. On the earth, the relative humidity of the air usually varies from 4% to 99%, depending on weather conditions.^[^ ^42^ ^]^ Change of water concentration through the air can swell/de‐swell a thin layer made of humidity‐sensitive material (e.g., agarose^[^ ^43^ ^]^) and induce effective bending deformation in mono‐ or bi‐layer construct.^[^ ^44^ ^]^ Humidity is generally considered as a source that lacks precision in control, due to random diffusing nature of water molecules that causes great challenges to manually pattern the humidity distribution in space. However, we note that a precise humidity gradient can be created by using the light field. The elevation of material temperature upon excitation of an optical beam can locally reduce the water content of the material in a high humidity chamber.^[^ ^45^ ^]^ Such light‐humidity actuation is observed to be with high spatial and temporal resolution, and ultra‐efficient in light actuation (intensity at the level of few mW cm^−2^).^[^ ^46^ ^]^ Thus, a synergistic use between photo‐ and humidity‐actuation could offer another novel approach to efficient flier control over a large spatial area.

In nature, dandelions use parachute‐like structures with 3D geometries to keep seeds aloft upon wind flow of arbitrary directions. The polymer assembly is restricted in 2D configuration, and exhibits a clear drawback of dispersal performance compared to its 3D counterpart in nature. Future study may include multiple layered actuating structures to induce more sophisticated 2D‐to‐3D shape‐morphing. The efficient integration between active elements (soft actuators) and passive structures (e.g., the pappus) within a miniature space is the foreseen challenge. It is practically useful to scale up the structure to a size about tens of centimetre. At a larger scale, the wind‐assisted gliders are expected to carry electronic devices for usefully applications. However, more robust actuator would be required to construct the porous architecture. On the other hand, it is fundamentally interesting to scale down the concept into microscopy. Two‐photon absorption laser fabrication allow micro construction of both passive and active elements, deemed as the suitable technique to create down‐scaled dispersal assembly.

Beyond dispersal, natural species implement gliding as another strategy to obtain efficient locomotion.^[^ ^47^ ^]^ In a steady gliding flight, the weight is balanced by the normal components (gravitational direction) of air drag and lift on the wings. Any change of wing geometry would vary the specific value of lift and drag coefficients, yielding a different gliding path, and asymmetry of the wing‐pair would tune the flight direction from straight to right‐/left‐turning. We believe that the precise control of LCE actuator in the dispersal flight proposed in this study, can be extended to a gliding device, in which a slight deflection of light‐responsive wings can significantly influence the specific trajectory of gliding. For both disperser and gliders, the deformation of the stimuli‐responsive material only provides a geometrical change of the passive flier. Due to the shape‐morphing, terminal velocity, drag coefficient and wind threshold for dispersal can be tuned, which significantly influences the locomotive behaviour during the passive flight. It should be noted that the motion of the actuator itself does not bring about air thrust – the actuator interacts with air flow passively. To attain an active flier, in which the wing flapping motion produces air thrust to induce propulsion in air, there still exist forbidden obstacles in the power density and actuation bandwidth of current actuating materials. The realization of an actively hovering flier with responsive materials will be the next challenge for material scientists, which would need the synergistic input from microroboticists.

To conclude, we have fabricated a dandelion‐inspired structure by assembling two bundles of fabric filament onto a soft photomechanical actuator. The assembly is able to perform either light‐ON‐to‐dispersal or light‐OFF‐to‐dispersal actions under a steady wind flow, depending on the bending direction of the actuator being fixed during the fabrication. The assembly generates a separated vortex ring downstream, similar as the natural dandelion seed. The vortex pattern can be reversibly controlled by photomechanically folding/unfolding the pappus structure. Such photo‐induced shape‐morphing can tune the terminal velocity and wind threshold for the dispersal, bringing about a quantitative control of the onset of lift‐off and landing actions. We demonstrate independent control of lift‐off of the structures, a hanging structure oscillating around a light beam, and light‐induced landing (local accumulation) in multiple free‐descending structures. These results suggest a new way to wirelessly control small devices that can passively fly in the sky.

## 4 Experimental Section

### Materials in Brief

1,4‐Bis‐[4‐(6‐acryloyloxyhexyloxy)benzoyloxy]‐2‐methylbenzene(99%, RM82) was purchased from SYNTHON Chemicals. 6‐amino‐1‐octanol and dodecylamine were purchased from TCI. Disperse Red 1 was purchased from Merck. All chemicals were used as received.

### Sample Fabrication

Liquid crystal cells were prepared by gluing two coated glass substrates, one was uni‐directionally rubbed polyvinyl alcohol (PVA, 5 wt.% in water, 3000 RPM, 1 min, baked at 90 °C for 10 min) for uniaxial alignment, the other with polyimide (PI, 3000 RPM, 1 min, baked at 180 °C for 20 min) for homeotropic alignment. 5 µm microspheres (Thermo Scientific) were used as the gap between two glass slides, in order to determine LCE film thickness. The liquid crystal mixture containing 0.3 mmol RM82, 0.115 mmol 6‐amino‐1‐octanol, 0.115 mmol dodecylamine, and 2.5 wt.% 2,2‐dimethoxy‐2‐phenylacetophenone (Irgacure 651) was melted at 85 °C and infiltrated into the cell via capillary at 85 °C and kept for 10 min. After cooling to 63 °C (1 °C min^−1^), the cells were stored in an oven at 63 °C for 24 h to conduct the aza‐Michael addition reaction (oligomerization). The samples were then irradiated with UV light (365 nm, 180 mW cm^−2^, 10 min) for polymerization. Finally, the cells were opened with a razor blade. 1 mg of Disperse Red 1 was spread onto the surface of the sample and diffused into the elastomer on a hot plate (100 °C for 10 min). The young's modulus of the LCE was 20 MPa at room temperature based on tensile testing.

### Fabrication of the Polymer Assembly

Fabric filaments were trimmed with 1.25, 1, 0.75 cm long. About 27 pieces of fabric filament were UV glued together into a semicircle. Two semicircles of bristle were then glued onto two ends of a rectangular LCE film to form the flier construct. LCE dimension: 4 mm × 2 mm × 0.005 mm, weight of 54 filaments (1 cm long): 0.66 mg, weight of LCE was 0.54 mg.

### Light Actuation

Collimated light beams from a continous solid state laser (ROITHNER, 2 W, 532 nm) or LED source (460 nm, CoolLED pE‐4000) were irradiated onto the LCE to induce deformation. The absorbed light power = light intensity (*I*) × LCE surface area. The *I* and sample dimension are detailed in the main text.

### Data Analysis

Tracking of position was done by using video analysis software (Kinovea). The data of PIV data were post‐processed by using the MATLAB toolbox PIVlab 1.41.^[^ ^48^ ^]^

### Force Measurement

A force sensor (F329, Novatech, 4 µN resolution) was used to measure the bending force. Details of the measurement see in Figure S11 (Supporting Information).

## Conflict of Interest

The authors declare no conflict of interest.

## Supporting information

Supporting Information

Supplemental Video 1

Supplemental Video 2

Supplemental Video 3

Supplemental Video 4

Supplemental Video 5

## Data Availability

The data that support the findings of this study are available from the corresponding author upon reasonable request.
