# Supplementary material for: Dandelion‐Inspired, Wind‐Dispersed Polymer‐Assembly Controlled by Light
Source: Adv Sci (Weinh). 2022 Dec 27;10(7):2206752. doi: 10.1002/advs.202206752 (PMC9982548; doi:10.1002/advs.202206752)
Supplement: Supplementary file 1 — Supporting Information [file ADVS-10-2206752-s002.PDF]

((Supporting Information can be included here using this template))

Supporting Information

Dandelion-inspired, wind-dispersed polymer-assembly controlled by light

Jianfeng Yang,[1] Hang Zhang,[2] Alex Berdin,[1] Wenqi Hu,[3] Hao Zeng[1]*

This PDF file includes:

Supplementary Figure S1 to S11

Methods
1. Wind tunnel constructure.
2. PIV measurement.
3. Comparison of SVR between natural and artificial assembly.

Captions for Supplementary Movies S1-S5

Supplementary Figures

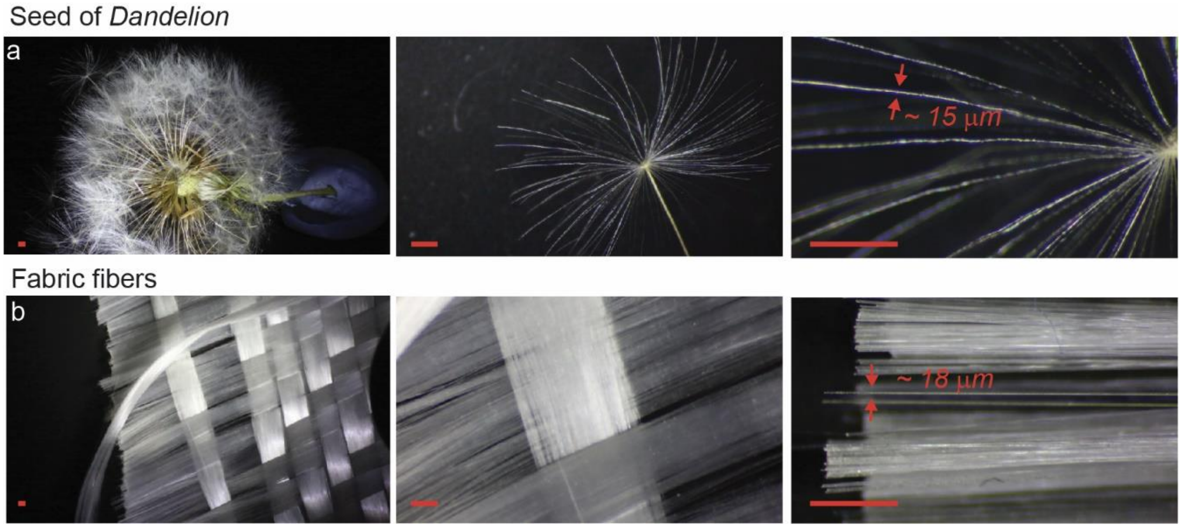

Figure S1. Photographic images of fabric filaments and natural bristle. (a) Meso-to-micro-scopic scale images of natural bristle at different magnifications. (b) Meso-to-micro-scopic images of fabric filaments. All scale bars are 1 mm.

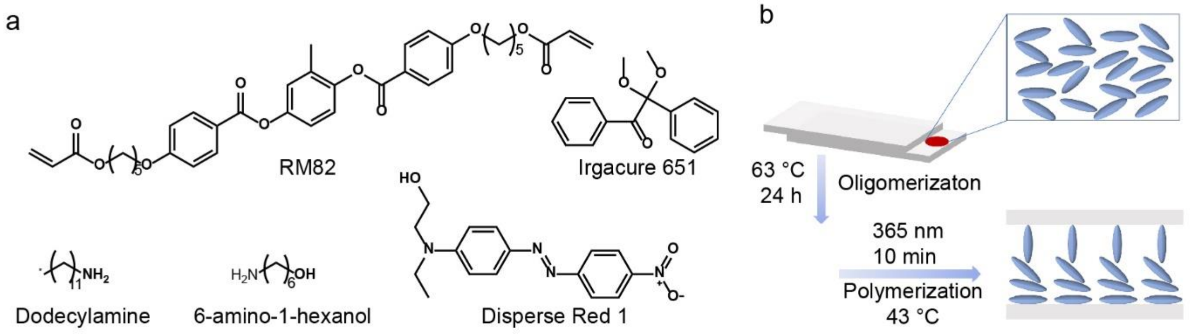

Figure S2. Synthetic steps of soft actuator. (a) Chemical structures of all molecules in use. Pre-cured mixture contains 0.3 mmol RM82, 0.115 mmol 6-amino-1-octanol, 0.115 mmol dodecylamine and 2.5 wt% Irgacure 651. (b) Schematic drawing of the LCE preparation process and molecular alignment.

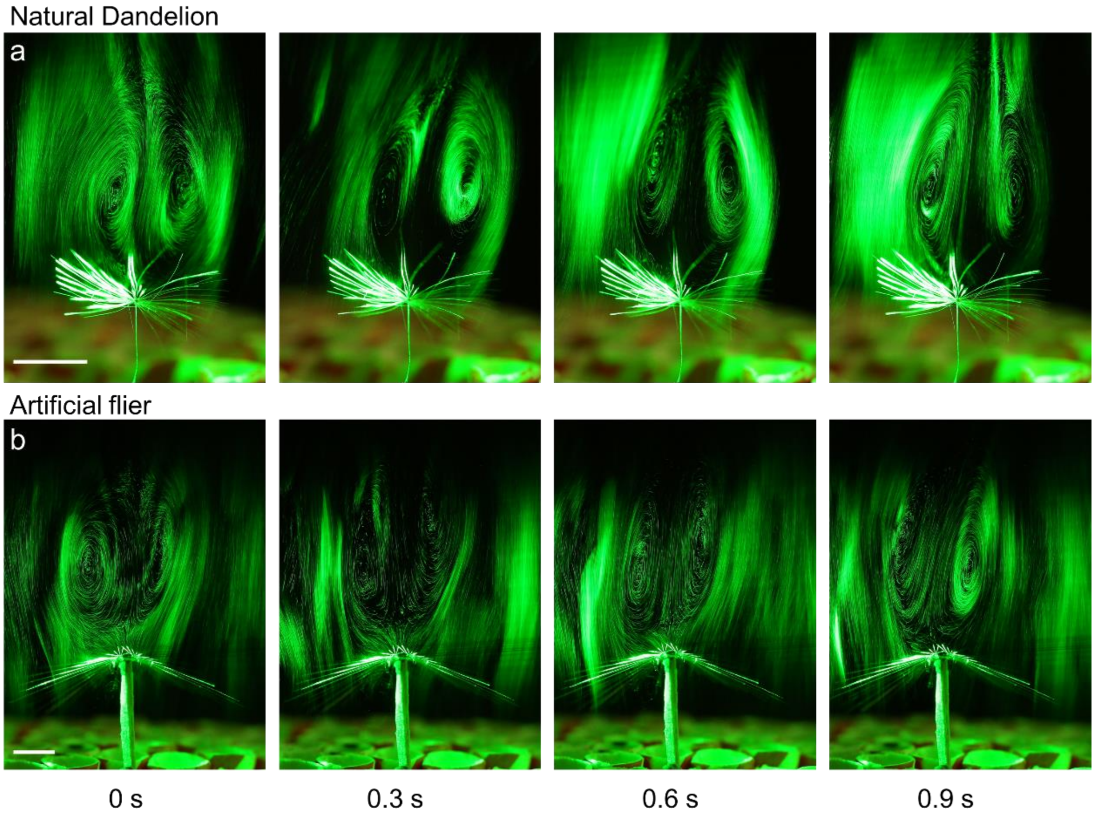

Figure S3. Stability of the vortex ring. Photo snaps of the vortex ring pattern downstream of a natural dandelion seed (a) and artificial flier (b). All scale bars are 5 mm.

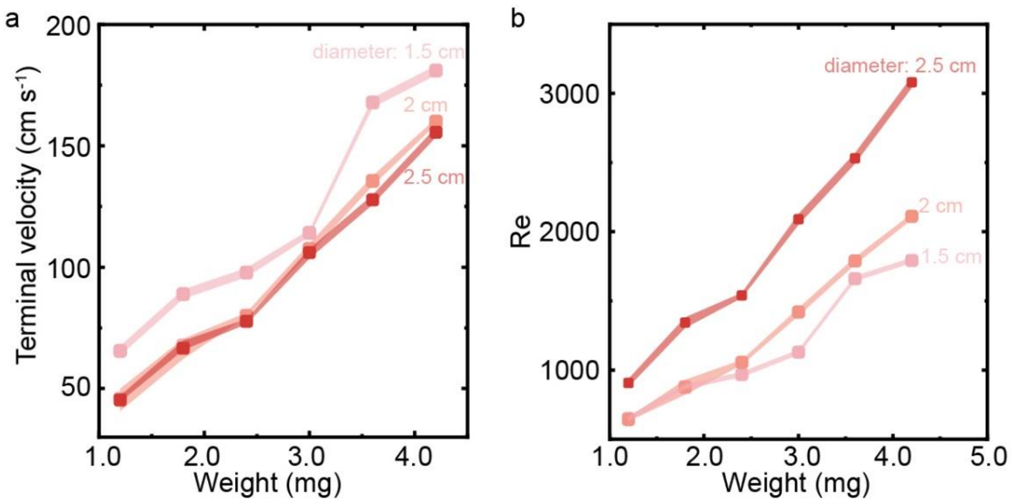

Figure S4. The terminal velocity and corresponding Reynolds number of fliers. The terminal velocity (a) and corresponding Reynolds number (*Re*) (b) of fliers with different diameters. The mass is tuned by trimming the seed segment or adding weight onto the flier. The error bars indicate standard deviation for n = 3 measurements.

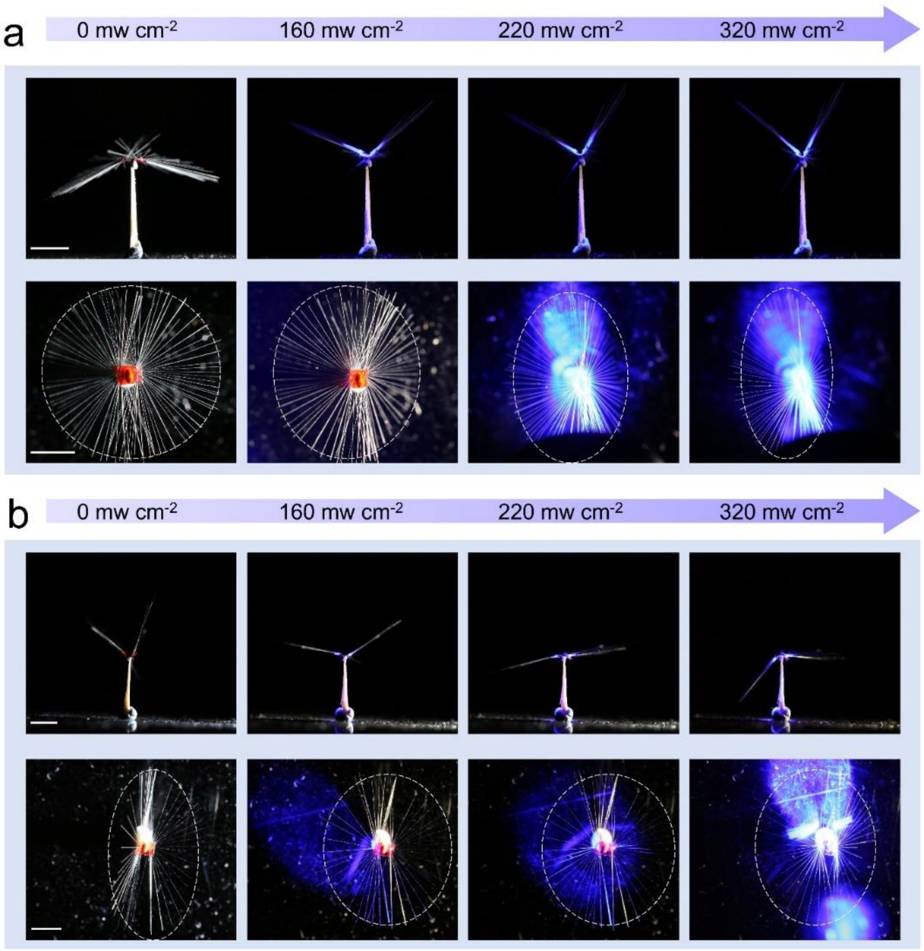

Figure S5. Light induced shape-morphing in artificial flier. Side-view and top-view photographs of a light-OFF-to-dispersal structure (a) and a light-ON-to-dispersal structure (b) upon increasing level of light irradiation. All scale bars are 0.5 cm.

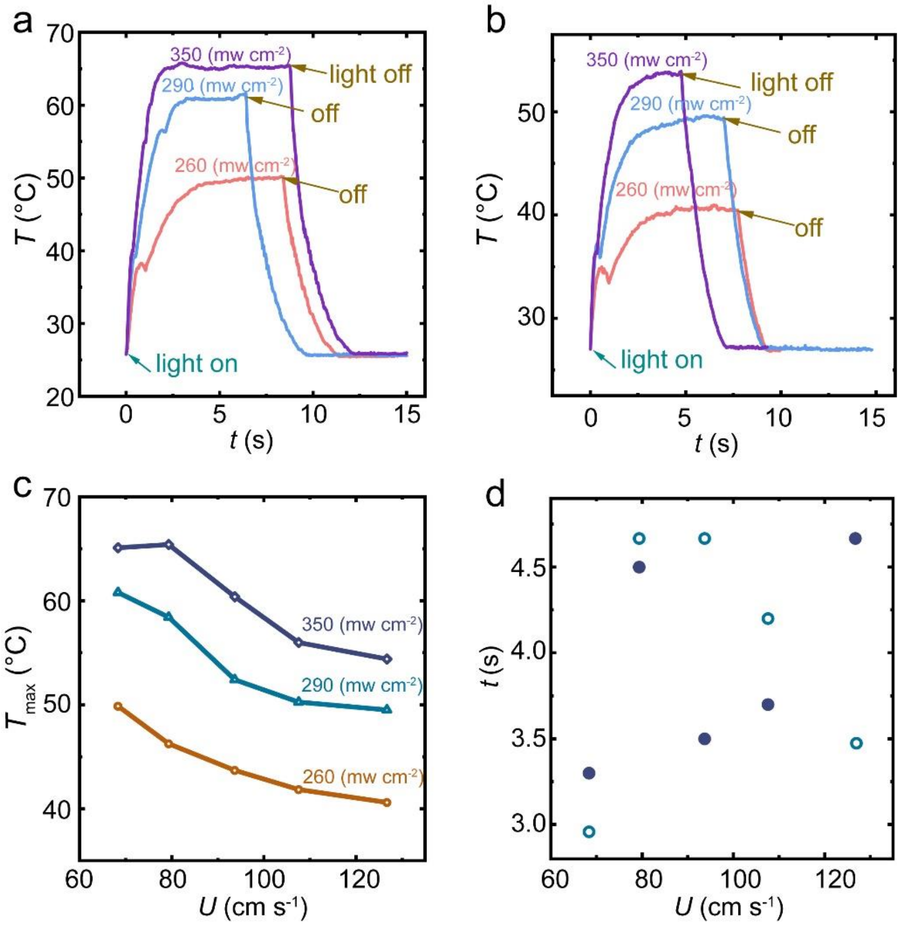

Figure S6. The photothermal kinetics upon different wind flows. Kinetics of light triggered bending upon 68 cm s^{-1} (a) and 127 cm s^{-1} (b) wind flow. (c) The maximum elevated temperature T_{max} upon different wind speeds (U) under different light intensities. (d) The time different (t) between light onset and the moment of reaching T_{max}, at different U. Light intensity: 290 mW cm^{-2} (bule circles) and 350 mW cm^{-2} (purple dots).

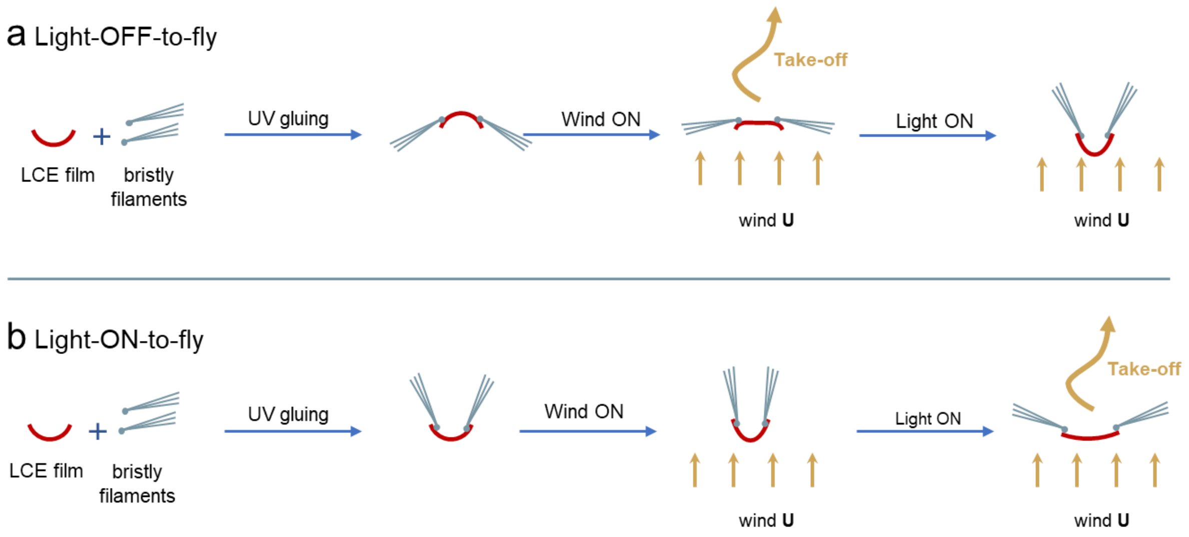

Figure S7. Schematic drawing of steps of fabrication. The LCE strip is initially bent after fabrication, due to the elevated temperature during photopolymerization. (a) For light-OFF-to-disperse structure, two bristly wings are glued on the LCE surface towards the actuation direction. Upon light cessation, the vertical wind drags exerting on the structure trends to reduce the curvature of the strip, thus flattening the wings. Such wind drag is not sufficient to bend the LCE strip to the opposite side. Upon light actuation, the strip bends to the opposite side, yielding a closure of the wings inside the wind flow. (b) For light-ON-to-disperse structure, two bristly wings are glued on the LCE surface opposite to the actuation direction. Without light illumination, the vertical wind drags further increase the curvature of the strip, yielding a folded structure inside the wing flow. Photomechanical deformation reverses the bending direction and flattens the wings. The structure thus opens its wings upon light irradiation.

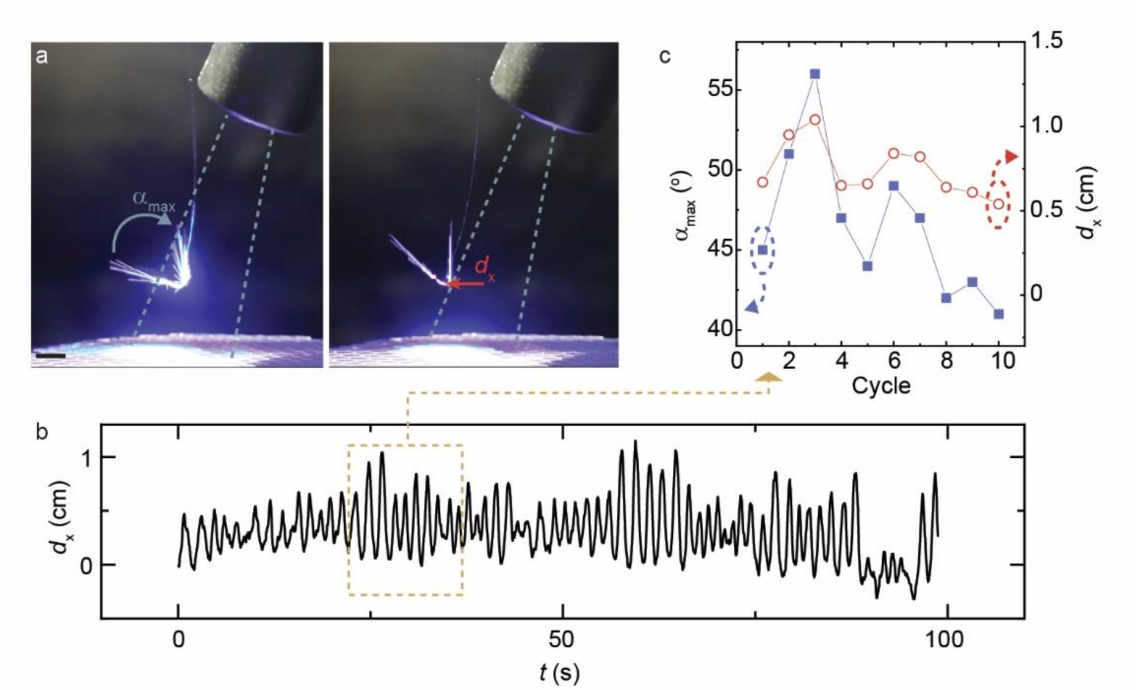

Figure S8. Feedback-based self-oscillation. (a) Photos of an oscillating structure inside and outside of a stationary light beam area. Inserts indicate the open-angle of the wing-pair *(*α, α_{max} is the maximum open-angle recorded in one oscillation cycle), and swing distance d_x along horizontal direction. Scale bar: 5 mm. (b) Track of position as a function of time upon constant excitation (460 nm, LCE source, spot size: 1.5 cm^{-2}, 320 mW cm^{-2}). (c) The variation of the α_{max} and d_x over ten oscillation cycles recorded within the time span highlighted by the dash line box in (b).

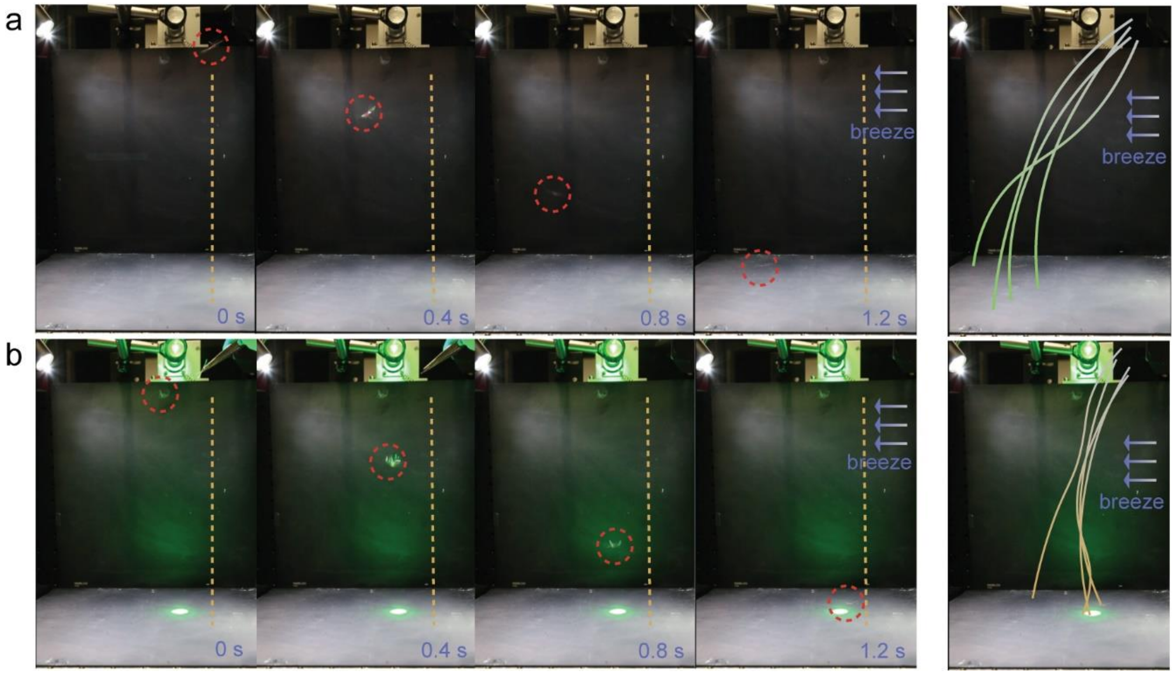

Figure S9. The free-descending experiment of an artificial flier. (a) Side-view snapshots of a structure being released to descend without laser excitation (left). Four trajectory examples of free descents (right). (b) Snapshots of a structure being released to descend upon laser excitation (left). Four examples of dropping trajectory that influenced by the light beam (right). Scale bar: 2 cm. Breeze: about 5 cm s^{-1}, horizontal direction.

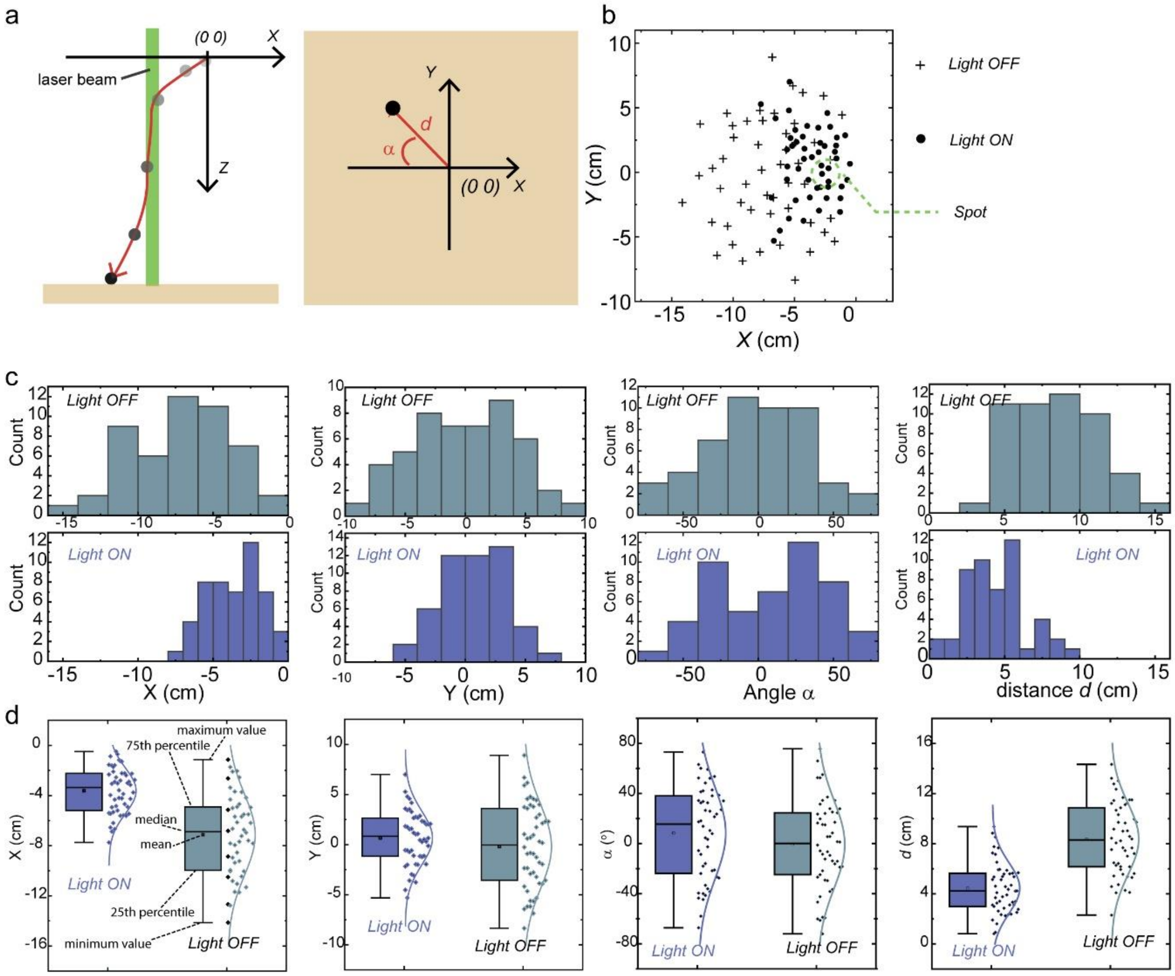

Figure S10. The statistics of landing point distribution. (a) Schematic drawing of the free-descent experiment: the structure is released at (0, 0, 0) coordinates (X-Y-Z), influenced by a laser beam (2 cm diameter) placed at (2 cm, 0, 0) along Z direction. Laser intensity is 1 W cm^{-2}. The landing point is recorded on the substrate at X-Y plane, with horizontally traveling distance, *d*, and directional angle, α. (b) Landing point distribution on the X-Y plane for 50 free-descent experiments at both light-ON and light-OFF conditions. (c) Counts of landing point in X- and Y- directions, α and *d*, upon light ON and OFF. (d) Statistics of landing point in X- and Y- directions, α and *d*, for both light excitation and cessation conditions.

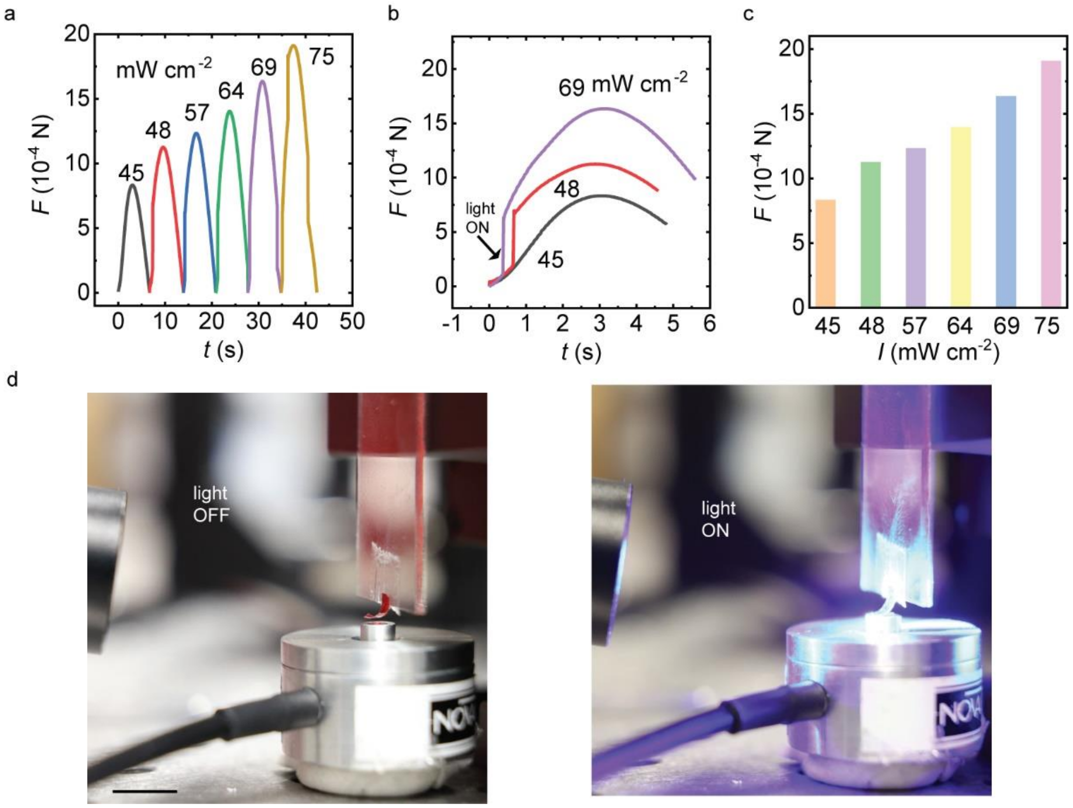

Figure S11. Light active force measurements. (a) The force generated by the bending of the LCE under different light intensities. (b) Kinetics of the force generation upon turning On the light source. (c) The maximum active force upon irradiation. (d) Photograph of the force measurement setup. Scale bar: 1 cm.

Methods

1. Wind tunnel construction.

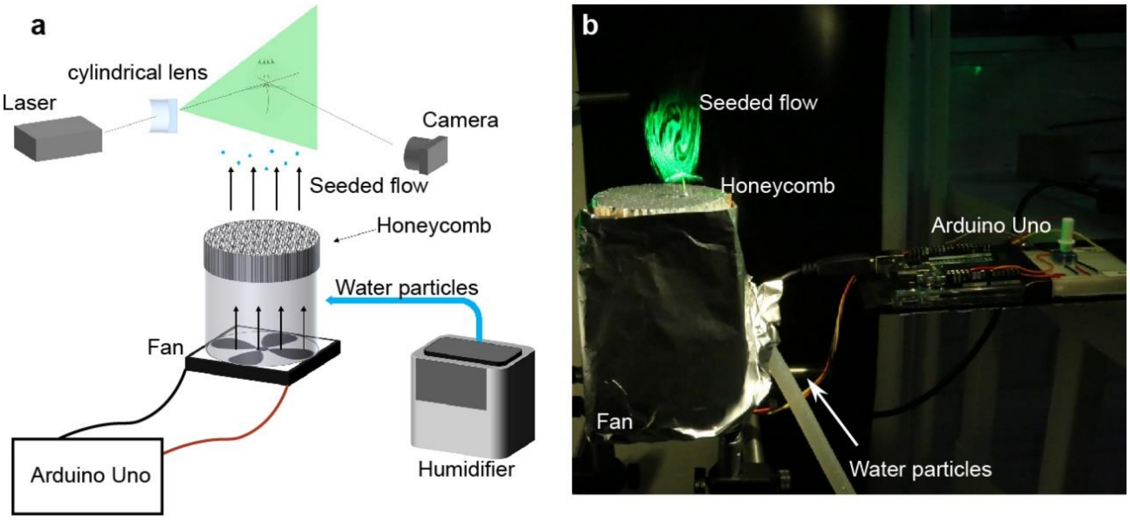

Figure S12. The setup for particle image velocimetry (PIV). (a) Schematic drawings of the wind tunnel, laser excitation and camera recording. (b) Photograph of experimental set up in laboratory.

A vertical wind tunnel was constructed to observe the airflow around a natural dandelion seed and artificial fliers. The tunnel is made by home-made cardboard tube, connected by glue. A fan (San Ace 52, 9GA0512P7A001) was placed at the bottom to generate airflow. The electric power of the fan is provided from power supply (PSP-2010, Yleiselektroniikka), the wind speed is controlled by Arduino Uno. The output of the tunnel was connected with meshes and a flow honeycomb (MAF Airflow Straightener Screen, 76 mm) straightener for regulating the air flow. A humidifier (Wilfa HU-400BC) was used to seed the air in the wind tunnel and visualize the airflow upon illumination of a laser beam (532 nm). Anemometer (AOPUTTRIVER AP-856A) was used to calibrate the wind velocity. The schematic drawing of the setup and optical photograph of the system are shown in Fig. S12.

2. PIV measurement.

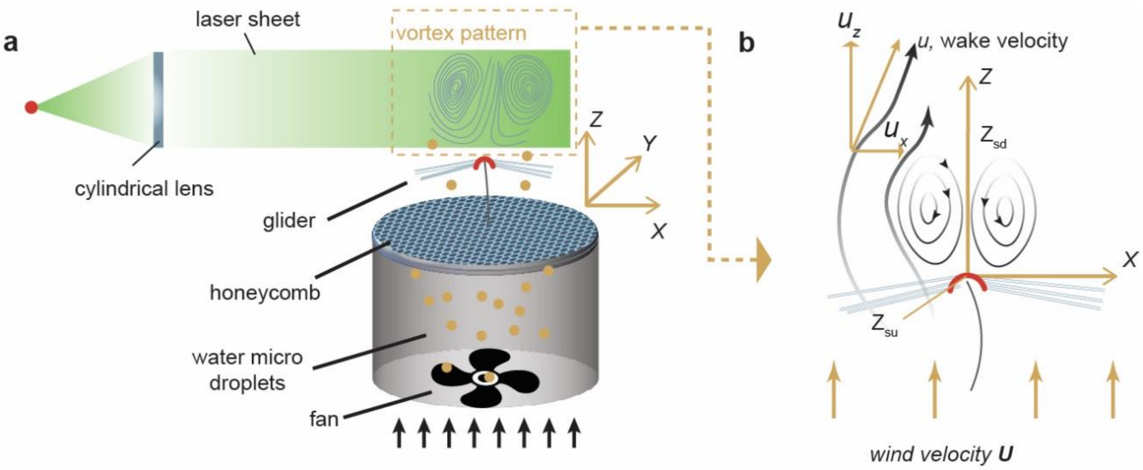

Figure S13. Particle image velocimetry (PIV) measurement. (a) Schematics of the PIV measurement setup. (b) Drawing of the coordinate system with the origin at the center of LCE actuator.

PIV is performed using a laser sheet to illuminate humidity-seeded air. The laser spot is reshaped into an ellipse by a cylindrical lens (Thorlabs, LJ4878, focus length: 75.0 mm). The laser sheet is propagating along X-direction to hit the water drop particles inside the wind flow. A Canon 5D Mark III camera with a 100 mm objective was used to capture the images and record movies. The sample rate is 25 Hz. The delta-T is 0.04 s. The data of PIV data were post-processed by using the MATLAB toolbox PIVlab 1.41. A fast Fourier window deformation technique was used: 136 × 136 pixel interrogation window in the first pass and a 68 × 68 pixel interrogation window in the second pass; Gaussian 2 × 3-point regression to estimate sub-pixel displacement. The measurement setup and coordinate system are shown in Fig. S13.

3. Comparison of SVR between natural and artificial assembly.

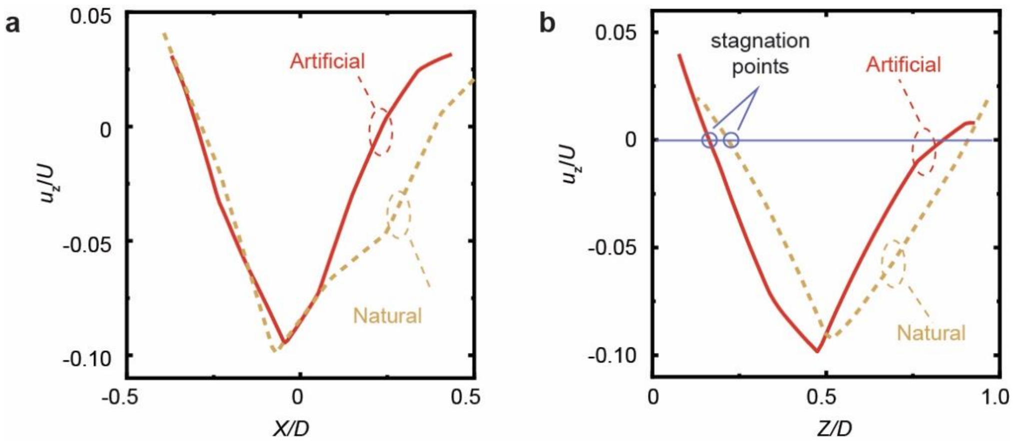

Figure S14. Particle image velocimetry (PIV) test between natural seed and artificial assembly. The ratio between the Z-axial velocities u_z and wind flow speed U measured along the lateral (X-axis) (a) and vertical (Z-axis) (b) directions.

We compare the aerodynamic behaviors of the natural and artificial assembly by measuring the flow downstream of their structures using PIV. The structures are vertically fixed at a position inside a wind tunnel with controlled flow speed U. The measurement setup is schematically shown in Figure S13, where an air flow seeded with water droplets (artificial fog) from a humidifier allows vortex visualization within a laser sheet probing the X-Z plane. In the flow downstream of the structure, the air wake velocity u (the flow downstream of the structure) can be decomposed into u_z along the vertical Z-axis and u_x along the lateral X-axis. The origin of the coordinate system (X, Y, Z) is selected to be the center of the LCE, as depicted in Figure S13b. Figure S14 plot the ratio between u_z and U, at different downstream positions along both lateral and vertical directions. The data shows a reverse flow of about 10% of U occurs in both natural and artificial structures. Importantly, the stagnation points of the upstream (z_{su}, determined by the position of stream where a zero velocity is observed) is always > 0 in both natural and artificial structure, indicating a separated vortex ring formation that is deemed important for passive dispersal mechanism.

Captions for Supplementary Movies

Movie S1. Separated vortex ring generation on natural dandelion seed and artificial structure. The seed of dandelion and artificial structure are vertically fixed at a position inside a wind tunnel with controlled flow speed. A humidifier was used to seed the air. The vortex is visualized by using a laser sheet illumination. The video is real time and 0.25× speed.

Movie S2. Dispersal behavior of an artificial structure controlled by light. By switching ON or OFF the light, the flier's take-off action can be performed. The video is real time. Wind flow: 0.7 m s^{-1} and 1.0 m s^{-1}, Light: 460 nm, 350 mW cm^{-2}.

Movie S3. Selective control of dispersal flight motion in multiple structures. A LED light beam is used to locally activate the structures on top of a wind tunnel. The selected structures then lift off and move away from the light spot. The video is real time. Wind flow: 1.0 m s^{-1}. Light: 460 nm, 350 mW cm^{-2}.

Movie S4. Self-oscillation by swinging around a light beam. The real time movie presents a self-swinging around a light beam governed by photo-mechano-air drag feedback. Wind flow: 0.7 m s^{-1}, Light: 460 nm, 210 W cm^{-2}. The video is real time.

Movie S5. Light influence on free-descending trajectory. The identical structure was released to descend without light and upon laser excitation. Laser: 532 nm, 1 W cm^{-2}. Horizontal wind flow is about 0.05 m s^{-1}. The video is real time.
